# Effects of 3-year denosumab treatment on hip structure in Japanese postmenopausal women and men with osteoporosis

**DOI:** 10.1016/j.bonr.2017.11.002

**Published:** 2017-11-14

**Authors:** Teruki Sone, Naohiro Kon, Kenneth W. Gaither, Naoki Okubo, Taisuke Osakabe, Yutaka Nakayama, Masao Fukunaga, Masako Ito, Toshitaka Nakamura

**Affiliations:** a Department of Nuclear Medicine, Kawasaki Medical School, 577 Matsushima, Kurashiki, Okayama 701-0192, Japan; b Medical Science Department, Daiichi Sankyo Co. Ltd., Tokyo, Japan; c Bioclinica, Inc., 11731 Northeast Glenn Widing Drive Portland, Oregon 97220, United States; d Biostatistics and Data Management Department, Daiichi Sankyo Co. Ltd., Tokyo, Japan; e Clinical Development Department, Daiichi Sankyo Co. Ltd., Tokyo, Japan; f Post-Marketing Regulatory Affairs Department, Daiichi Sankyo Co. Ltd., Tokyo, Japan; g Kawasaki Medical School, 577 Matsushima, Kurashiki, Okayama 701-0192, Japan; h Center for Diversity and Inclusion, Nagasaki University, 1-14 Bunkyo-machi, Nagasaki 852-8521, Japan; i Touto Sangenjaya Rehabilitation Hospital, 1-24-3 Sangenjaya, Setagaya-ku, Tokyo 154-0024, Japan

**Keywords:** RANK, receptor activator of nuclear factor kappa-B, HSA, hip structural analysis, BMD, bone mineral density, FREEDOM, Fracture Reduction Evaluation of Denosumab in Osteoporosis every 6 Months, DIRECT, Denosumab fracture Intervention Randomized placebo Controlled Trial, DXA, dual-energy X-ray absorptiometry, PBO/DMAb, placebo/denosumab, DMAb/DMAb, denosumab/denosumab, ED, endocortical diameter, OD, outer diameter, CoTh, cortical thickness, CSA, cross sectional area, CSMI, cross sectional moment of inertia, SM, section modulus, BR, buckling ratio, Osteoporosis, Denosumab, RANK ligand, Hip structural analysis, Japanese, Bone quality

## Abstract

Denosumab, a human monoclonal antibody against RANK ligand, is shown to have strong anti-fracture effects in Japanese osteoporosis patients. However, there have been no data showing actions on Japanese bone architecture. Here we show that denosumab continuously improves several geometrical parameters calculated by hip structural analysis for 3 years. Compared to placebo, denosumab significantly increased bone mineral density, cortical thickness and cross sectional area in all of the three analyzed areas: the narrow neck, intertrochanter and femoral shaft. The subsequent derived mechanical parameters, cross-sectional moment of inertia, section modulus and buckling ratio, were also improved by denosumab. In addition, the improvement of these parameters was also observed in the patients that had switched from placebo to denosumab treatment. The present study suggests the structural evidence explaining the strong anti-fracture efficacy of denosumab and its significant effects on cortical bone in Japanese.

## Highlights

• Denosumab improves several HSA parameters including BMD and cortical thickness.
• The improvement of HSA parameters continues at least 3 years.
• The improvement of HSA parameters was observed in the patients that had switched from placebo to denosumab.

## 1 Introduction

Osteoporosis is a systemic skeletal disorder characterized by compromised bone strength predisposing a person to an increased risk of fracture (Black and Rosen, 2016). Typical traits of the disease are cortical thinning and a deterioration of trabecular microstructure. Osteoporotic fractures lead to severe consequences, such as hospitalization, immobility for long periods, surgical treatment or significant increase of mortality risk (Bolland et al., 2010, Tarantino et al., 2016a).

The risk of fractures is estimated by bone strength, which is considered to be primarily due to bone mineral density (BMD) and bone quality (Lorentzon and Cummings, 2015). Bone strength is improved by treatment with several pharmacologic agents, which are classified as anabolic or antiresorptive agents (Black and Rosen, 2016, Russell, 2015, Appelman-Dijkstra and Papapoulos, 2015). The anabolic agent parathyroid hormone (PTH) stimulates osteoblasts to form new bone by activating its receptors. On the other hand, antiresorptives including bisphosphonates and denosumab, target osteoclast-mediated bone resorption. Bisphosphonates have been used in clinical medicine for > 40 years. After binding to bone matrix, bisphosphonates are internalized into osteoclasts. Bisphosphonates act as analogs of pyrophosphate enzyme substrates and interfere with several biochemical processes, which results in suppression of osteoclast activities. Denosumab is the first biologic therapy approved to treat osteoporosis. Denosumab inhibits bone resorption by binding to and inactivating RANK ligand (RANKL), a key mediator of osteoclast formation, maturation and activation.

In the pivotal phase 3 trial FREEDOM, subcutaneous administration of 60 mg denosumab every 6 months significantly reduced the risk of new vertebral, nonvertebral and hip fractures compared to placebo in predominantly postmenopausal Caucasian women with osteoporosis (Cummings et al., 2009). The study was extended to a total of 10 years of treatment, and the results showed continuous BMD increase, low fracture incidence and good safety profile (Bone et al., 2017). The continuous BMD increase by denosumab is clinically important, because the effects of bisphosphonate treatment appear to plateau earlier, after about 3 years (Russell, 2015). In addition, several studies revealed advantages of denosumab compared to bisphosphonates (Scott, 2014, Zebaze et al., 2014, Benjamin et al., 2016). For example, denosumab increases BMD and reduces markers of bone turnover to a significantly greater extent than bisphosphonates (Brown et al., 2009). The strong effects of denosumab are also observed in those who had switched from alendronate to denosumab treatment (Kendler et al., 2010).

In Japanese osteoporosis patients, the efficacy of denosumab was evaluated by the study called DIRECT, a double-blind, placebo-controlled trial with an open-label weekly 35 mg alendronate arm. The DIRECT study showed that 2-year treatment of denosumab significantly reduced incidence of new vertebral fracture by 74.0% compared to placebo (Nakamura et al., 2014). The following 12-month extension study showed that 3-year treatment of denosumab was associated with low fracture rates, persistent bone turnover marker reductions and continuous BMD increase (Sugimoto et al., 2015).

In addition to the BMD change, analyses of effects on bone quality parameters, including bone geometry, are important for understanding the basis of the strong anti-fracture efficacy of denosumab (Beck et al., 2008, Austin et al., 2012). To this point, hip structural analysis using dual-energy X-ray absorptiometry (DXA) data is useful to evaluate hip geometry (Beck and Broy, 2015), as demonstrated to be a good predictor of hip fracture risk by prospective studies (Rivadeneira et al., 2007, Kaptoge et al., 2008). The previous HSA study showed denosumab improves structural parameters for 2 years in Caucasian osteoporosis patients (Beck et al., 2008), supporting the good results shown by the FREEDOM study. Because several reports have indicated significant differences in hip axis length between Asian and Caucasian (Broy et al., 2015, Cummings et al., 1994), it is important in clinical pharmacology to clarify effects of denosumab on hip structure in Japanese. Thus, we investigated denosumab effects on the HSA parameters using data of the DIRECT study. In addition, the present study shows 3-year effect of denosumab on the HSA parameters for the first time.

## 2 Materials and methods

### 2.1 Study participants

DIRECT study consisted of a 2-year randomized, double-blind, placebo-controlled phase and a 1-year open-label extension phase, in which all subjects received denosumab. The study participants were described previously (Nakamura et al., 2014). The present analysis used DXA data of the total hip obtained at baseline (before the administration), 6, 12, 24 and 36 months after the start of the treatment with denosumab or placebo. While images for 952 patients were obtained for placebo/denosumab (PBO/DMAb) group and denosumab/denosumab (DMAb/DMAb) group in DIRECT study, it is impossible to analyze the images for 265 patients because the scanners used were not applicable to HSA. Thus, the images for 687 patients were used for the present HSA study.

### 2.2 Analysis of hip geometry

Quality control and analysis of DXA scans were performed by trained DXA technicians from Bioclinica (Oregon, USA) in a blinded manner. Bioclinica DXA technicians are annually tested to within 1.5% rmsCV of a gold standard set of images. Hip geometry was analyzed using Hologic Apex DXA software version 13.5 (Madison, WI) in accordance with a standardized HSA analysis protocol (Beck, 2002). The DXA technologists defined a global region of interest around the proximal femur and placed three analysis regions in their defined positions on the femur image within the DXA analysis software. The narrow neck region was placed perpendicular to the neck axis and at the narrowest section of the neck (Supplementary Fig. 1). The intertrochanteric region was placed in order to bisect the neck shaft angle, defined as the intersection of the neck and shaft midlines, and should rest above the lesser trochanter. The shaft region was placed perpendicular to the shift midline and 2 cm below the distal edge of the lesser trochanter. All regions were adjusted to include at least 1 but optimally 5 pixels of soft tissue in either end of the region. At follow-up visits, the three analysis regions were placed in the same way in order to match the baseline analysis as closely as possible. If the follow-up scan positioning was dissimilar to such a degree that the analysis regions could not be placed similarly to baseline, the scan was excluded.

The HSA program derives the geometry from lines of pixel values (mass profiles) traversing the bone at each of the three regions. All cross-sectional geometries are calculated from mass profile distributions converted to linear thickness by dividing each pixel value by the effective mineral density of fully mineralized tissue. Measurements include: BMD as average pixel value in the profile, bone outer diameter (OD), endocortical diameter (ED), mineralized bone cross-sectional area (CSA), average cortical thickness (CoTh), cross-sectional moment of inertia (CSMI) as BMD times square of the distance from the center of mass, section modulus (SM) as CSMI divided by D_max_ (maximum distance between the center of the mass and the outer cortex), buckling ratio (BR) as the ratio of bone diameter to average cortical thickness, and D_max_. Neck and shaft sections are modeled as circular annuli with 60% and 100% of the CSA in the cortex respectively. The intertrochanteric region is modeled as an elliptical annulus with an anteroposterior diameter as the measured shaft width and 70% of the CSA in the cortex. Models are used for cortex estimates and buckling ratio but not for OD, CSA, CSMI and SM.

### 2.3 Statistical analysis

Statistical analysis included all randomized subjects who received at least one dose of investigational product and had a baseline and at least one post-baseline scan. The images for 687 patients were evaluable for HSA in the present study. Percent change from baseline in HSA parameters at each time point were analyzed using one-sample *t*-test. Comparisons between the treatment groups for the percent changes in HSA parameters at each time point were performed using a two-sample t-test. Missing values were imputed using the last observation carried forward method. The mean values and 95% confidence intervals over time were graphically presented.

## 3 Results

In the images obtained in DIRECT study for 952 patients, the images for 687 patients applicable to HSA were used in the present study. Baseline characteristics (sex, age, proximal femoral BMD and femoral neck BMD) in the 687 patients were similar to those in the total 952 participants (Table 1) (Nakamura et al., 2014). Geometric parameters in hip structure are depicted in Table 2. These background parameters were well matched between PBO/DMAb and DMAb/DMAb. A summary of all HSA parameters analyzed in the present study, difference between PBO/DMAb and DMAb/DMAb in mean percent change from baseline at month 12 is depicted in Table 3. HSA parameters were analyzed in three ways: 1) change from baseline at each time point in PBO/DMAb, 2) change from baseline at each time point in DMAb/DMAb and 3) comparison of the changes at each time point between PBO/DMAb and DMAb/DMAb. Results of each HSA parameter were described in detail below.

**Table 1:** Baseline characteristics of subjects.

Characteristics	Placebo/Denosumab (*N* = 327)	Denosumab/Denosumab (N = 321)
Sex, n (%)		
Female	306 (93.6)	304 (94.7)
Male	21 (6.4)	17 (5.3)
Age (years)	69.6 ± 7.6	70.1 ± 7.5
Years since menopause^a^	20.3 ± 9.0	21.0 ± 9.0
Body height (cm)	151.2 ± 6.4	150.8 ± 6.6
Body weight (kg)	50.9 ± 7.9	51.0 ± 7.0
BMI (kg/m^2^)	22.3 ± 3.0	22.5 ± 2.8
BMD T-score		
Lumbar spine (L1-L4)^b^	− 2.73 ± 0.89	− 2.75 ± 0.89
Total hip	− 1.99 ± 0.68	− 2.03 ± 0.79
Femoral neck	− 2.36 ± 0.66	− 2.42 ± 0.67

Data shown are mean ± SD unless otherwise specified.

a Subject numbers in PBO/DMAb and DMAb/DMAb are 306 and 303, respectively.

b Subject numbers in PBO/DMAb and DMAb/DMAb are 297 and 308, respectively.

**Table 2:** Baseline geometric parameters in hip structural analysis.

Characteristics	Placebo/Denosumab (*n* = 327)	Denosumab/Denosumab (*n* = 321)
Narrow neck		
BMD (g/cm^2^)	0.65 ± 0.08	0.64 ± 0.09
ED (cm)	2.93 ± 0.25	2.94 ± 0.25
OD (cm)	3.18 ± 0.25	3.18 ± 0.24
CoTh (cm)	0.12 ± 0.02	0.12 ± 0.02
CSA (cm^2^)	1.96 ± 0.28	1.93 ± 0.27
D_max_ (cm)	1.39 ± 0.12	1.39 ± 0.11
CSMI (cm^4^)	1.56 ± 0.43	1.54 ± 0.41
SM (cm^3^)	0.86 ± 0.18	0.85 ± 0.17
BR	14.85 ± 2.68	15.12 ± 2.86

Intertrochanter		
BMD (g/cm^2^)	0.64 ± 0.09	0.63 ± 0.10
ED (cm)	4.69 ± 0.37	4.69 ± 0.34
OD (cm)	5.21 ± 0.37	5.20 ± 0.32
CoTh (cm)	0.26 ± 0.04	0.26 ± 0.05
CSA (cm^2^)	3.17 ± 0.49	3.14 ± 0.51
D_max_ (cm)	2.18 ± 0.19	2.18 ± 0.18
CSMI (cm^4^)	8.03 ± 2.24	7.87 ± 1.98
SM (cm^3^)	2.63 ± 0.60	2.60 ± 0.58
BR	11.95 ± 2.14	12.18 ± 2.73

Femoral shaft		
BMD (g/cm^2^)	1.16 ± 0.17	1.16 ± 0.18
ED (cm)	2.05 ± 0.29	2.04 ± 0.32
OD (cm)	2.87 ± 0.21	2.87 ± 0.21
CoTh (cm)	0.41 ± 0.07	0.41 ± 0.08
CSA (cm^2^)	3.16 ± 0.47	3.15 ± 0.46
D_max_ (cm)	1.39 ± 0.11	1.39 ± 0.10
CSMI (cm^4^)	2.51 ± 0.61	2.48 ± 0.57
SM (cm^3^)	1.67 ± 0.31	1.66 ± 0.28
BR	3.75 ± 0.87	3.77 ± 0.96

Data shown are mean ± SD. BMD, bone mineral density; ED, endocortical diameter; OD, outer diameter; CoTh, cortical thickness; CSA, cross sectional area; D_max_, maximum distance between the center of the mass and the outer cortex; CSMI, cross sectional moment of inertia; SM, section modulus; BR, buckling ratio.

**Table 3:** Difference between PBO/DMAb and DMAb/DMAb in mean percent change from baseline at month 12.

	Narrow neck	Intertrochanter	Femoral shaft
BMD	3.24 (2.65, 3.83)⁎⁎	4.12 (3.45, 4.78)⁎⁎	2.55 (2.00, 3.11)⁎⁎
ED	− 0.55 (− 0.96, − 0.14)⁎	− 0.77 (− 1.15, − 0.39)⁎⁎	− 1.44(− 2.02, − 0.86)⁎⁎
OD	− 0.25 (− 0.61, 0.11)	− 0.30 (− 0.64, 0.05)	− 0.07(− 0.34, 0.20)
CoTh	3.44 (2.82, 4.07)⁎⁎	4.14 (3.41, 4.88)⁎⁎	3.10 (2.41, 3.80)⁎⁎
CSA	2.99 (2.39, 3.59)⁎⁎	3.84 (3.18, 4.49)⁎⁎	2.48 (1.96, 2.99)⁎⁎
D_max_	0.33 (− 0.16, 0.82)	1.12 (0.57, 1.66)⁎⁎	0.19 (− 0.15, 0.53)
CSMI	2.21 (1.10, 3.32)⁎	3.65 (2.40, 4.90)⁎⁎	2.14 (1.43, 2.86)⁎⁎
SM	2.96 (2.04, 3.87)⁎⁎	4.95 (3.72, 6.19)⁎⁎	2.43 (1.76, 3.10)⁎⁎
BR	− 4.01 (− 4.95, − 3.07)⁎⁎	− 5.15 (− 5.99, − 4.31)⁎⁎	− 3.33 (− 4.23, − 2.43)⁎⁎

Data shown are mean (95% CI).

⁎ *p* < 0.01.

⁎⁎ *p* < 0.0001.

### 3.1 Bone mineral density (BMD)

**Fig. 1:**
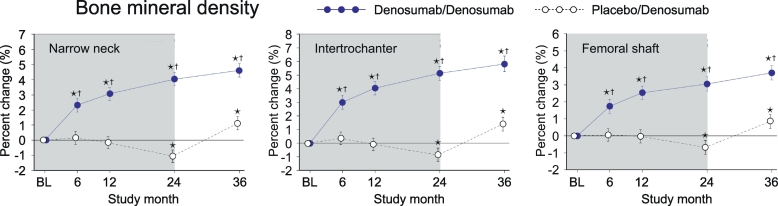
Bone mineral density calculated by HSA. Data are mean with 95% confidence interval. Number of data is 327 or 321 for placebo/denosumab group (white) or denosumab/denosumab group (blue), respectively. Data of baseline (BL), 6, 12 and 24 months were obtained by double blind study phase (depicted in shadow) for the treatment of denosumab or placebo. Data of 36 months were obtained by the subsequent 12-month open-label study phase, in which both groups (blue and white) were treated with denosumab. Asterisk indicates *p* < 0.05 compared to BL value in each group. Dagger in denosumab/denosumab group indicates *p* < 0.05 compared to placebo/denosumab value in each time point. (For interpretation of the references to colour in this figure legend, the reader is referred to the web version of this article.)

In PBO/DMAb group, BMD significantly decreased from the baseline level during PBO treatment in all regions examined, the narrow neck, intertrochanter and femoral shaft (Fig. 1, white circles). Importantly, the decreased values of BMD during PBO treatment were increased by the treatment with denosumab, as observed in the significantly higher value at month 36 than the baseline level. In DMAb/DMAb group, BMD showed continuous increase from month 0 to 36 (Fig. 1, blue circles). When DMAb/DMAb was compared to PBO/DMAb, the values of DMAb/DMAb showed significantly higher value than those of PBO/DMAb at all time points in all regions.

### 3.2 Endocortical diameter (ED)

**Fig. 2:**
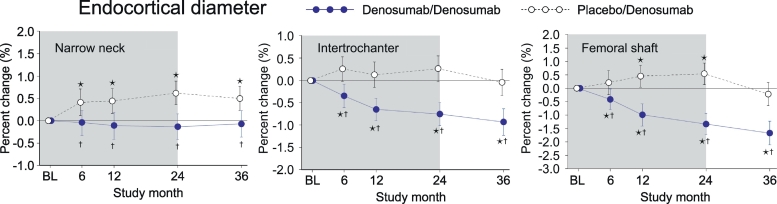
Endocortical diameter calculated by HSA. Data are mean with 95% confidence interval. Number of data is 327 or 321 for placebo/denosumab group or denosumab/denosumab group, respectively. Asterisk indicates *p* < 0.05 compared to baseline (BL) value in each group. Dagger in denosumab/denosumab group indicates *p* < 0.05 compared to placebo/denosumab value in each time point.

In PBO/DMAb group, ED increased in the narrow neck and femoral shaft, whereas no significant change was observed in the intertrochanter (Fig. 2, white circles). In DMAb/DMAb group, ED showed no change in the narrow neck, whereas continuous decrease was observed from month 0 to 36 in the intertrochanter and femoral shaft (Fig. 2, blue circles). When DMAb/DMAb was compared to PBO/DMAb, the values of DMAb/DMAb showed significantly lower than those of PBO/DMAb at all time points in all regions.

### 3.3 Outer diameter (OD)

**Fig. 3:**
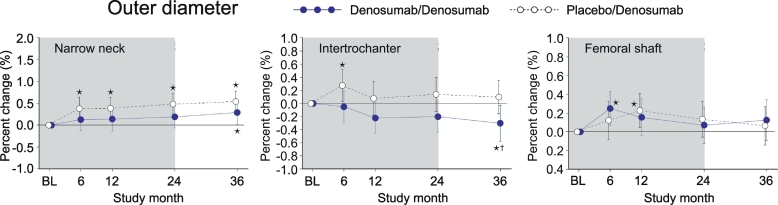
Outer diameter calculated by HSA. Data are mean with 95% confidence interval. Number of data is 327 or 321 for placebo/denosumab group or denosumab/denosumab group, respectively. Asterisk indicates *p* < 0.05 compared to baseline (BL) value in each group. Dagger in denosumab/denosumab group indicates p < 0.05 compared to placebo/denosumab value in each time point.

In PBO/DMAb group, OD showed continuous increase at month 6, 12 and 24 in the narrow neck, whereas the increase was observed only at month 6 or 12 in the intertrochanter or femoral shaft, respectively (Fig. 3, white circles). In DMAb/DMAb group, significant increase from the baseline level was observed at month 36 in the narrow neck and at month 6 in femoral shaft, whereas a decrease was observed at month 36 in the intertrochanter (Fig. 3, blue circles). When DMAB/DMAb was compared to PBO/DMAb, the value of DMAb/DMAb at month 36 in the intertrochanter was significantly lower than that of PBO/DMAb.

### 3.4 Cortical thickness (CoTh)

**Fig. 4:**
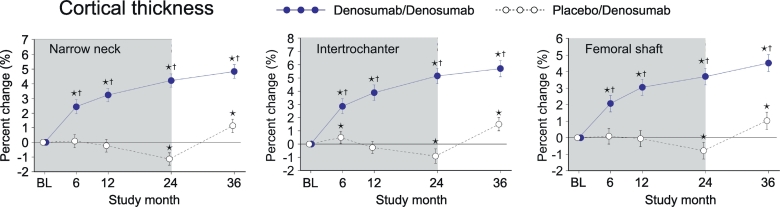
Cortical thickness calculated by HSA. Data are mean with 95% confidence interval. Number of data is 327 or 321 for placebo/denosumab group or denosumab/denosumab group, respectively. Asterisk indicates *p* < 0.05 compared to baseline (BL) value in each group. Dagger in denosumab/denosumab group indicates *p* < 0.05 compared to placebo/denosumab value in each time point.

In PBO/DMAb group, CoTh significantly decreased at month 24 compared to the baseline level in all regions (Fig. 4, white circles). Importantly, the decreased values of CoTh were increased by the treatment with denosumab, as observed in the significantly higher values at month 36 than the baseline level. In DMAb/DMAb group, CoTh showed continuous increase from month 0 to 36 (Fig. 4, blue circles). When DMAb/DMAb was compared to PBO/DMAb, the values of DMAb/DMAb showed significantly higher than those of PBO/DMAb group at all time points in all regions.

### 3.5 Cross sectional area (CSA)

**Fig. 5:**
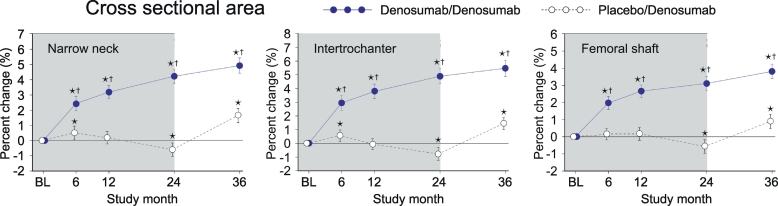
Cross sectional area calculated by HSA. Data are mean with 95% confidence interval. Number of data is 327 or 321 for placebo/denosumab group or denosumab/denosumab group, respectively. Asterisk indicates *p* < 0.05 compared to baseline (BL) value in each group. Dagger in denosumab/denosumab group indicates *p* < 0.05 compared to placebo/denosumab value in each time point.

In PBO/DMAb group, CSA significantly decreased at month 24 compared to the baseline level in all regions (Fig. 5, white circles). Importantly, the decreased values of CSA were increased by the treatment with denosumab, as observed in the significantly higher value at month 36 than the baseline level. In DMAb/DMAb group, CSA showed continuous increase from month 0 to 36 (Fig. 5, blue circles). When DMAb/DMAb was compared to PBO/DMAb, the values of DMAb/DMAb showed significantly higher than those of PBO/DMAb group at all time points in all regions.

### 3.6 D_max_ (maximum distance between the center of the mass and the outer cortex)

**Fig. 6:**
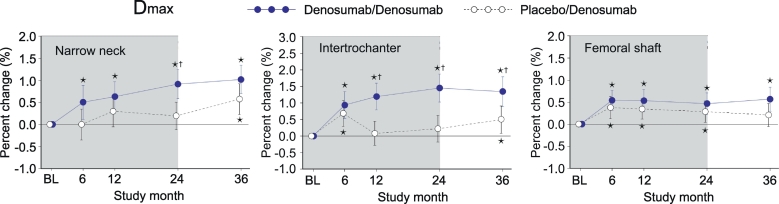
D_max_ calculated by HSA. Data are mean with 95% confidence interval. Number of data is 327 or 321 for placebo/denosumab group or denosumab/denosumab group, respectively. Asterisk indicates *p* < 0.05 compared to baseline (BL) value in each group. Dagger in denosumab/denosumab group indicates *p* < 0.05 compared to placebo/denosumab value in each time point.

In PBO/DMAb group, no significant change was observed in the narrow neck, whereas the increase at month 6 or 6–24 was observed in the intertrochanter or femoral shaft, respectively (Fig. 6, white circles). In DMAb/DMAb group, D_max_ showed continuous increase from month 0 to 36 (Fig. 6, blue circles). When DMAb/DMAb was compared to PBO/DMAb, the significantly high values of DMAb/DMAb were observed at month 24 in the narrow neck or at 12–36 in the intertrochanter.

### 3.7 Cross sectional moment of inertia (CSMI)

**Fig. 7:**
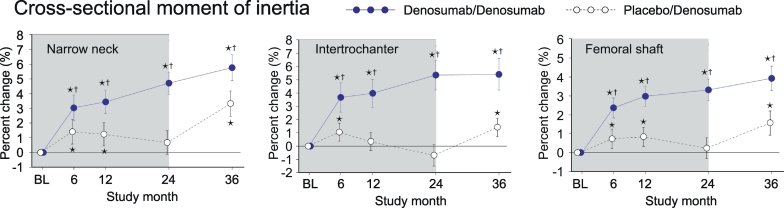
Cross-sectional moment of inertia calculated by HSA. Data are mean with 95% confidence interval. Number of data is 327 or 321 for placebo/denosumab group or denosumab/denosumab group, respectively. Asterisk indicates *p* < 0.05 compared to baseline (BL) value in each group. Dagger in denosumab/denosumab group indicates *p* < 0.05 compared to placebo/denosumab value in each time point.

In PBO/DMAb group, CSMI initially increased at month 6, whereas the values at month 24 were not significantly different from the baseline values in the all regions (Fig. 7, white circles). The values were increased by the treatment with denosumab, as observed in the significantly higher values at month 36 compared to the baseline level. In DMAb/DMAb group, CSMI showed continuous increase from month 0 to 36 (Fig. 7, blue circles). When DMAb/DMAb was compared to PBO/DMAb, the values of DMAb/DMAb showed significantly higher than those of PBO/DMAb group at all time points in all regions.

### 3.8 Section modulus (SM)

**Fig. 8:**
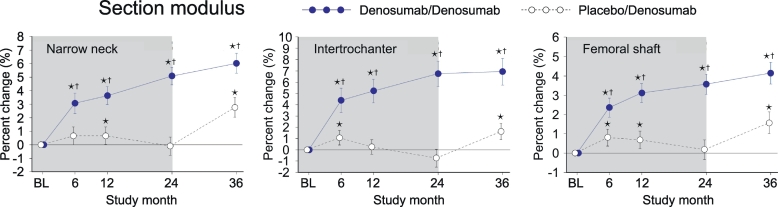
Section modulus calculated by HSA. Data are mean with 95% confidence interval. Number of data is 327 or 321 for placebo/denosumab group or denosumab/denosumab group, respectively. Asterisk indicates *p* < 0.05 compared to baseline (BL) value in each group. Dagger in denosumab/denosumab group indicates *p* < 0.05 compared to placebo/denosumab value in each time point.

In PBO/DMAb group, SM showed transient increase at month 6 and 12, whereas the value at month 24 was not significantly different from the baseline level in across all regions (Fig. 8, white circles). The values were increased by the treatment with denosumab, as observed in the significantly higher values at month 36 than the baseline level. In DMAb/DMAb group, SM showed continuous increase from month 0 to 36 (Fig. 8, blue circles). When DMAb/DMAb was compared to PBO/DMAb, the values of DMAb/DMAb showed significantly higher than those of PBO/DMAb group at all time points in all regions.

### 3.9 Buckling ratio (BR)

**Fig. 9:**
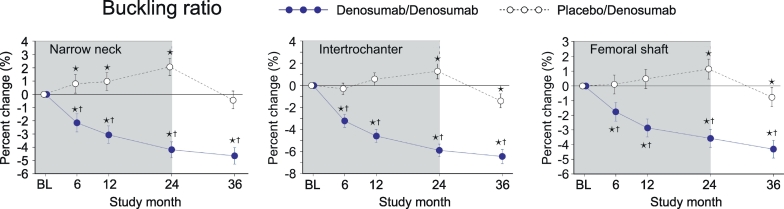
Buckling ratio calculated by HSA. Data are mean with 95% confidence interval. Number of data is 327 or 321 for placebo/denosumab group or denosumab/denosumab group, respectively. Asterisk indicates *p* < 0.05 compared to baseline (BL) value in each group. Dagger in denosumab/denosumab group indicates *p* < 0.05 compared to placebo/denosumab value in each time point.

In PBO/DMAb group, BR showed a trend to increase until 24 months. In the narrow neck, the values at month 6, 12 and 24 were significantly higher than baseline (Fig. 9, white circles). In the intertrochanter and femoral shaft, the values at month 24 were significantly higher. On the other hand, the increased values were reduced by the treatment with denosumab in all regions. Consistently, BR values of DMAb/DMAb group showed continuous decrease from month 0 to 36 (Fig. 9, blue circles). When DMAb/DMAb was compared to PBO/DMAb, the values of DMAb/DMAb showed significantly lower than those of PBO/DMAb group at all time points in all regions.

## 4 Discussion and conclusions

The present study revealed the continuous 3-year effects of denosumab on HSA parameters in Japanese postmenopausal women and men with osteoporosis. We also obtained data showing temporal change of the geometric parameters for 2 years in Japanese osteoporosis patients from the analysis of 327 patients in the placebo group. Significant deterioration was observed in this group in BMD, CoTh, CSA and BR at month 24 in all regions. The trend in the placebo group was also observed in the previous study using 111 patients for 72 weeks (Sone et al., 2014).

Denosumab significantly improves BMD, CoTh, CSA, D_max_, CSMI, SM and BR in all of the three areas (narrow neck, intertrochanter and femoral shaft) compared to placebo (Fig. 1, Fig. 4, Fig. 5, Fig. 6, Fig. 7, Fig. 8, Fig. 9). We also observed significant changes of ED from baseline in the intertrochanter and shaft (Fig. 2). These improving effects on hip structural parameters are similar to the previous studies of Caucasians for 2 years (Beck et al., 2008, Bone et al., 2008), and the effects are demonstrated to continue for 3 years in the present study. Importantly, effects of denosumab last for long periods of time as demonstrated by the recent study showing continuous increase of BMD for 10 years (Bone et al., 2017).

Comparison of osteoporosis drugs is important to understand their characteristics. Previous Japanese HSA study revealed that teriparatide increases BMD in the narrow neck and intertrochanter by 2–3%, whereas no significant change is observed in the shaft (Sone et al., 2014). On the other hand, denosumab increased BMD of the narrow neck, intertrochanter or shaft by 3, 4 or 2.5%, respectively (Fig. 1). Similarly, significant improvement of CoTh, CSA or BR in the shaft was observed only in denosumab treatment (Fig. 4, Fig. 5, Fig. 9) (Sone et al., 2014). These results indicate that denosumab has strong effects on the shaft. The shaft is a unique region that can be recognized as 100% cortical bone (Beck, 2002). Consistent with the present results, strong effects of denosumab on cortical bone are also shown by 3-dimensional analytical methods such as high resolution peripheral quantitated computed tomography (HR-pQCT) or 3D bone mapping study in Caucasians (Seeman et al., 2010, Poole et al., 2015). The strong action on cortical bone may be one of mechanisms for the strong anti-fracture effect of denosumab, because cortical bone has an important role in the axial load-bearing capacity of long bones (Tarantino et al., 2016b).

Denosumab is demonstrated to have stronger effects than alendronate on BMD improvement (Brown et al., 2009). Consistently, the previous HSA study revealed that denosumab shows greater effects on several HSA parameters than alendronate at the intertrochanteric and shaft sites in Caucasians (Beck et al., 2008). Although the present study includes no direct comparative data, the previous Japanese alendronate study is informative (Takada et al., 2011). A similar trend is observed also in Japanese. The potent and continuous effects can be explained by two possible mechanisms. First, bisphosphonates act on only one step, activation of mature osteoclasts, whereas denosumab acts on multiple steps, formation, maturation and activation of osteoclasts (Lacey et al., 2012). Second, bisphosphonates require bone surfaces to exert the suppressive effects on osteoclast activity, whereas denosumab exerts the action without binding to bone matrix (Zebaze et al., 2014, Beck et al., 2008). Cortical bone has a much lower ratio of surface to volume than trabecular bone. The structural difference may cause relatively less binding of bisphosphonates to cortical bone than trabecular bone.

There are limitations in HSA methodology because the analysis is based on a 2-dimensional DXA image and requires several methodological assumptions (Beck and Broy, 2015). First, since HSA methods assumes a fixed proportion of cortical and cancellous bone mass to calculate ED, the estimate of ED and its derived measurements such as CoTh and BR are influenced when bone mass changes in cortical and cancellous bone are not proportional. In this point, however, the shaft is least subject to the limitations of HSA, because the site is primarily cortical bone and more regularly shaped. Thus, it is an important observation that several HSA parameters showed significant change from baseline in the shaft (Fig. 1, Fig. 4, Fig. 5, Fig. 6, Fig. 7, Fig. 8, Fig. 9).

Another limitation is the possible error due to the assumption of fixed bone tissue mineralization density in HSA. Antiresorptive drugs increase average bone tissue mineralization, which may cause an overestimate of CSA, CSMI, and SM measured by HSA. Increase in bone tissue mineralization may also cause an overestimate of OD measured by HSA by partial volume effect error in determining bone edge. However, the present results showed no continuous increase of OD during the treatment with denosumab (Fig. 3). Thus the result apparently shows that there are no false increases of OD.

The present study reveals 3-year effect of denosumab on the geometrical parameters of hip bone structure. The improving effects suggest underlying mechanism for anti-fracture effect of denosumab (Nakamura et al., 2014, Sugimoto et al., 2015). Further characterization of denosumab effects on bone quality, such as microstructure, is important for clinical pharmacology and skeletal pathology in order to manage bone fracture risk in osteoporosis.

The following is the supplementary data related to this article.

Supplementary Fig. 1 The narrow neck, intertrochanter and shaft regions were set as described in the section of materials and methods. Supplementary Fig. 1

## Author contribution

T. Sone, T. Nakamura, M. Ito and M. Fukunaga provided opinions as medical specialists. N. Kon, T. Sone, T. Nakamura, Y. Nakayama, T. Osakabe and N. Okubo planned the project and discussed the data. K. W. Gaither analyzed X-ray image data and N. Okubo statistically analyzed the results. N. Kon wrote the manuscript with supports by K. W. Gaither and N. Okubo. The manuscript was reviewed by the all authors. N. Kon and Y. Nakayama led this company initiated study.

## References

[bb0005] Appelman-Dijkstra N.M., Papapoulos S.E. (2015). Modulating bone resorption and bone formation in opposite directions in the treatment of postmenopausal osteoporosis. Drugs.

[bb0010] Austin M., Yang Y.C., Vittinghoff E., Adami S., Boonen S., Bauer D.C. (2012). Relationship between bone mineral density changes with denosumab treatment and risk reduction for vertebral and nonvertebral fractures. J. Bone Miner. Res..

[bb0015] Beck T.J. (2002). Morgan RH. Hip structural Analysis (HSA) Program -BMD and Structural Geometry Methodology-. The 3rd National Health and Nutrition Examination Survey Dataset.

[bb0020] Beck T.J., Broy S.B. (2015). Measurement of hip geometry-technical background. J. Clin. Densitom..

[bb0025] Beck T.J., Lewiecki E.M., Miller P.D., Felsenberg D., Liu Y., Ding B. (2008). Effects of denosumab on the geometry of the proximal femur in postmenopausal women in comparison with alendronate. J. Clin. Densitom..

[bb0030] Benjamin B., Benjamin M.A., Swe M., Sugathan S. (2016). Review on the comparison of effectiveness between denosumab and bisphosphonates in post-menopausal osteoporosis. Osteoporos. Sarcopenia.

[bb0035] Black D.M., Rosen C.J. (2016). Clinical practice. Postmenopausal osteoporosis. N. Engl. J. Med..

[bb0040] Bolland M.J., Grey A.B., Gamble G.D., Reid I.R. (2010). Effect of osteoporosis treatment on mortality: a meta-analysis. J. Clin. Endocrinol. Metab..

[bb0045] Bone H.G., Bolognese M.A., Yuen C.K., Kendler D.L., Wang H., Liu Y. (2008). Effects of denosumab on bone mineral density and bone turnover in postmenopausal women. J. Clin. Endocrinol. Metab..

[bb0050] Bone H.G., Wagman R.B., Brandi M.L., Brown J.P., Chapurlat R., Cummings S.R. (2017). 10 years of denosumab treatment in postmenopausal women with osteoporosis: results from the phase 3 randomised FREEDOM trial and open-label extension. Lancet Diabetes Endocrinol..

[bb0055] Brown J.P., Prince R.L., Deal C., Recker R.R., Kiel D.P., de Gregorio L.H. (2009). Comparison of the effect of denosumab and alendronate on BMD and biochemical markers of bone turnover in postmenopausal women with low bone mass: a randomized, blinded, phase 3 trial. J. Bone Miner. Res..

[bb0060] Broy S.B., Cauley J.A., Lewiecki M.E., Schousboe J.T., Shepherd J.A., Leslie W.D. (2015). Fracture risk prediction by non-BMD DXA measures: the 2015 ISCD official positions part 1: hip geometry. J. Clin. Densitom..

[bb0065] Cummings S.R., Cauley J.A., Palermo L., Ross P.D., Wasnich R.D., Black D. (1994). Racial differences in hip axis lengths might explain racial differences in rates of hip fracture. Study of osteoporotic fractures research group. Osteoporos. Int..

[bb0070] Cummings S.R., San Martin J., McClung M.R., Siris E.S., Eastell R., Reid I.R. (2009). Denosumab for prevention of fractures in postmenopausal women with osteoporosis. N. Engl. J. Med..

[bb0075] Kaptoge S., Beck T.J., Reeve J., Stone K.L., Hillier T.A., Cauley J.A. (2008). Prediction of incident hip fracture risk by femur geometry variables measured by hip structural analysis in the study of osteoporotic fractures. J. Bone Miner. Res..

[bb0080] Kendler D.L., Roux C., Benhamou C.L., Brown J.P., Lillestol M., Siddhanti S. (2010). Effects of denosumab on bone mineral density and bone turnover in postmenopausal women transitioning from alendronate therapy. J. Bone Miner. Res..

[bb0085] Lacey D.L., Boyle W.J., Simonet W.S., Kostenuik P.J., Dougall W.C. (2012). Bench to bedside: elucidation of the OPG-RANK-RANKL pathway and the development of denosumab. Nat. Rev. Drug Discov..

[bb0090] Lorentzon M., Cummings S.R. (2015). Osteoporosis: the evolution of a diagnosis. J. Intern. Med..

[bb0095] Nakamura T., Matsumoto T., Sugimoto T., Hosoi T., Miki T., Gorai I. (2014). Clinical trials express: fracture risk reduction with denosumab in Japanese postmenopausal women and men with osteoporosis: denosumab fracture intervention randomized placebo controlled trial (DIRECT). J. Clin. Endocrinol. Metab..

[bb0100] Poole K.E., Treece G.M., Gee A.H., Brown J.P., McClung M.R., Wang A. (2015). Denosumab rapidly increases cortical bone in key locations of the femur: a 3D bone mapping study in women with osteoporosis. J. Bone Miner. Res..

[bb0105] Rivadeneira F., Zillikens M.C., De Laet C.E., Hofman A., Uitterlinden A.G., Beck T.J. (2007). Femoral neck BMD is a strong predictor of hip fracture susceptibility in elderly men and women because it detects cortical bone instability: the Rotterdam study. J. Bone Miner. Res..

[bb0110] Russell R.G. (2015). Pharmacological diversity among drugs that inhibit bone resorption. Curr. Opin. Pharmacol..

[bb0115] Scott L.J. (2014). Denosumab: a review of its use in postmenopausal women with osteoporosis. Drugs Aging.

[bb0120] Seeman E., Delmas P.D., Hanley D.A., Sellmeyer D., Cheung A.M., Shane E. (2010). Microarchitectural deterioration of cortical and trabecular bone: differing effects of denosumab and alendronate. J. Bone Miner. Res..

[bb0125] Sone T., Ito M., Fukunaga M., Tomomitsu T., Sugimoto T., Shiraki M. (2014). The effects of once-weekly teriparatide on hip geometry assessed by hip structural analysis in postmenopausal osteoporotic women with high fracture risk. Bone.

[bb0130] Sugimoto T., Matsumoto T., Hosoi T., Miki T., Gorai I., Yoshikawa H. (2015). Three-year denosumab treatment in postmenopausal Japanese women and men with osteoporosis: results from a 1-year open-label extension of the denosumab fracture intervention randomized placebo controlled trial (DIRECT). Osteoporos. Int..

[bb0135] Takada J., Katahira G., Iba K., Yoshizaki T., Yamashita T. (2011). Hip structure analysis of bisphosphonate-treated Japanese postmenopausal women with osteoporosis. J. Bone Miner. Metab..

[bb0140] Tarantino U., Rao C., Tempesta V., Gasbarra E., Feola M. (2016). Hip fractures in the elderly: the role of cortical bone. Injury.

[bb0145] Tarantino U., Rao C., Tempesta V., Gasbarra E., Feola M. (2016). Hip fractures in the elderly: the role of cortical bone. Injury.

[bb0150] Zebaze R.M., Libanati C., Austin M., Ghasem-Zadeh A., Hanley D.A., Zanchetta J.R. (2014). Differing effects of denosumab and alendronate on cortical and trabecular bone. Bone.

