# Supplementary figures and images for: Effects of 3-year denosumab treatment on hip structure in Japanese postmenopausal women and men with osteoporosis
Source: Bone Rep. 2017 Nov 14;7:164–71. doi: 10.1016/j.bonr.2017.11.002 (PMC5701790; doi:10.1016/j.bonr.2017.11.002)

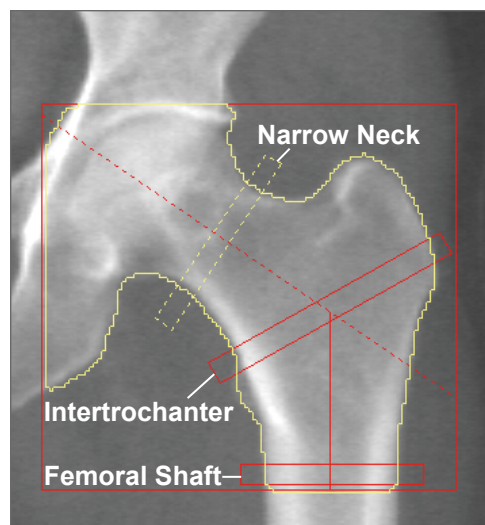

Figure-S1

Supplement: Supplementary Fig. 1 — The narrow neck, intertrochanter and shaft regions were set as described in the section of materials and methods. [file mmc1.pdf]
